# Supplementary material for: Explaining distortions in metacognition with an attractor network model of decision uncertainty
Source: PLoS Comput Biol. 2021 Jul 26;17(7):e1009201. doi: 10.1371/journal.pcbi.1009201 (PMC8341696; doi:10.1371/journal.pcbi.1009201)
Supplement: S1 Appendix — (DOCX) [file pcbi.1009201.s001.docx]

**S1 Appendix**

**Model fit to experiment 2 data from Rouault et al. (2018)**

Experiment 2 (n=497) is identical to experiment 1 in all but three aspects. First, Rouault et al. (2018) used a staircase (calibration) procedure to fix participants’ perceptual performance [1,2]. The staircase procedure was two-down one-up, with equal step sizes. Step-sizes (in logspace) were: 0.4 for first 5 trials, 0.2 for next 5, 0.1 for the rest of the task. The starting point was 4.2. Each participant completed 25 practice trials at the beginning of the task to minimise the burn-in period. Second, participants reported their confidence on a 6-point confidence scale which ranged from 1= guessing to 6=certainly correct). Third, pre- and post-task global confidence ratings were omitted from experiment 2.

Similar to our analysis of Experiment 1, we first fitted our neural circuit model to subjects’ choices and response times, but not confidence reports (see Methods). The results show (see Figs AA and AB) that our model again accounts for both average patterns of choice accuracy and response times, and individual differences across participants (Figs AC and AD).

To fit the model to subjects’ confidence reports, we first simulated our neural circuit model with the parameters fitted to subjects’ choices and response times from experiment 2, and applied distribution matching to map the model’s simulated uncertainty levels onto subjects’ retrospective confidence reports (see Methods in main text). The results (Fig B) show that the model accounts for the complex relationship between decision confidence and task difficulty, and the results hold after conditioning confidence reports on the outcome of the trial (i.e. correct vs. error).


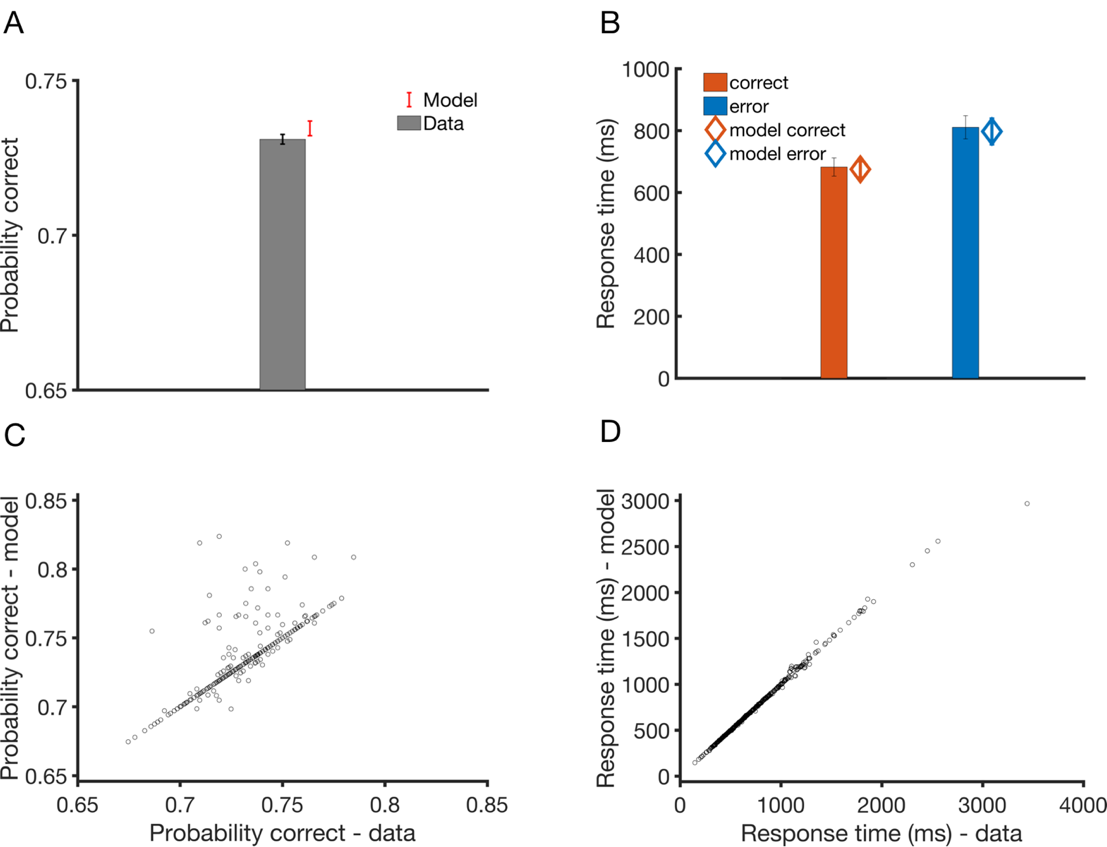


**Fig A.** **Model accounts for subjects’ perceptual performance in experiment 2**. **A.** Choice accuracy averaged across all participants from experiment 2. Model fit (red) accounts for the data (grey bar). **B.** Response times averaged across all participants from experiment 2, split by correct (orange) and error (blue) responses. Slower (faster) responses in case of error (correct) responses are observed both in the model (markers) and data (bars). Error bars indicate 95% confidence interval. **C.** Scatter plot of observed vs. simulated mean response times and **(D)** accuracy for each of the 497 subjects. Random seed is reset after each simulation during fitting procedure and for the purposes of generating figures C and D.


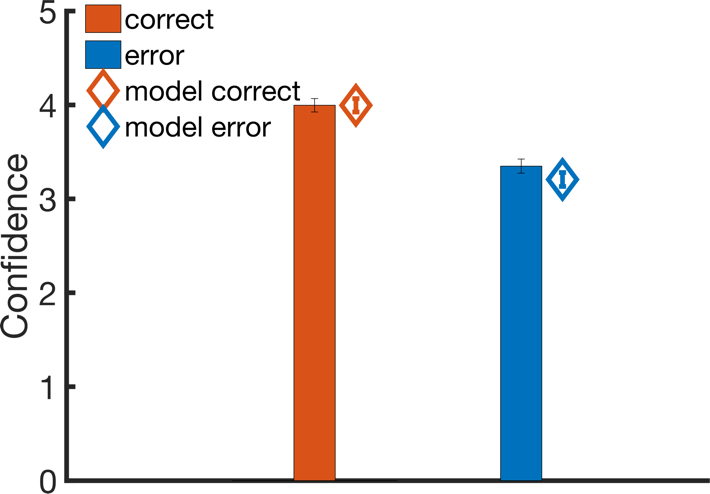


**Fig B. Model accounts for subjects’ confidence reports in experiment 2.** Confidence reports averaged across all participants from experiment 2 data (bars) and model (markers). Blue (orange) markers: Error (correct) responses. Error bars indicate 95% confidence interval.

1. Garcı́a-Pérez MA. Forced-choice staircases with fixed step sizes: asymptotic and small-sample properties. Vision Research. 1998;38(12):1861-81. doi: 10.1016/s0042-6989(97)00340-4.

2. Fleming SM, Weil RS, Nagy Z, Dolan RJ, Rees G. Relating introspective accuracy to individual differences in brain structure. Science. 2010;329(5998):1541-3. doi: 10.1126/science.1191883. PubMed PMID: 20847276.
