# Supplementary material for: Explaining distortions in metacognition with an attractor network model of decision uncertainty
Source: PLoS Comput Biol. 2021 Jul 26;17(7):e1009201. doi: 10.1371/journal.pcbi.1009201 (PMC8341696; doi:10.1371/journal.pcbi.1009201)
Supplement: S2 Appendix — (DOCX) [file pcbi.1009201.s002.docx]

**S2 Appendix**

**Relationships between psychiatric symptoms and model parameters in Experiment 2**

In experiment 2, additional questionnaires were added (Eating Attitudes Test, Apathy Evaluation Scale, and Alcohol Use Disorders Identification Test), and the Generalized Anxiety Disorder 7-item scale was replaced by the State Trait Anxiety Inventory (STAI) questionnaire. Additionally, participants completed a short IQ test (International Cognitive Ability Resource) [1].

We found no significant association between self-reported scores (or cross-cutting factors derived from these scores, see below) and model parameters (Fig A). We considered whether this lack of significance in experiment 2 may have been due to the smaller variance in difficulty (due to the staircase procedure) leading to inferences on uncertainty modulation being less constrained by the data than in experiment 1 (see Fig C, left). To explore this further, we attempted to recover the fitted parameters to both experiment 1 and 2 data and found that the fit to experiment 1 data is indeed more stable (Figs B and C) – potentially due to the difference in difficulty variation (see Fig D). We note however that qualitatively, similar symptom scores (e.g. depression, anxiety) that were negatively related to uncertainty modulation in experiment 1 were also negatively related to uncertainty modulation in experiment 2.


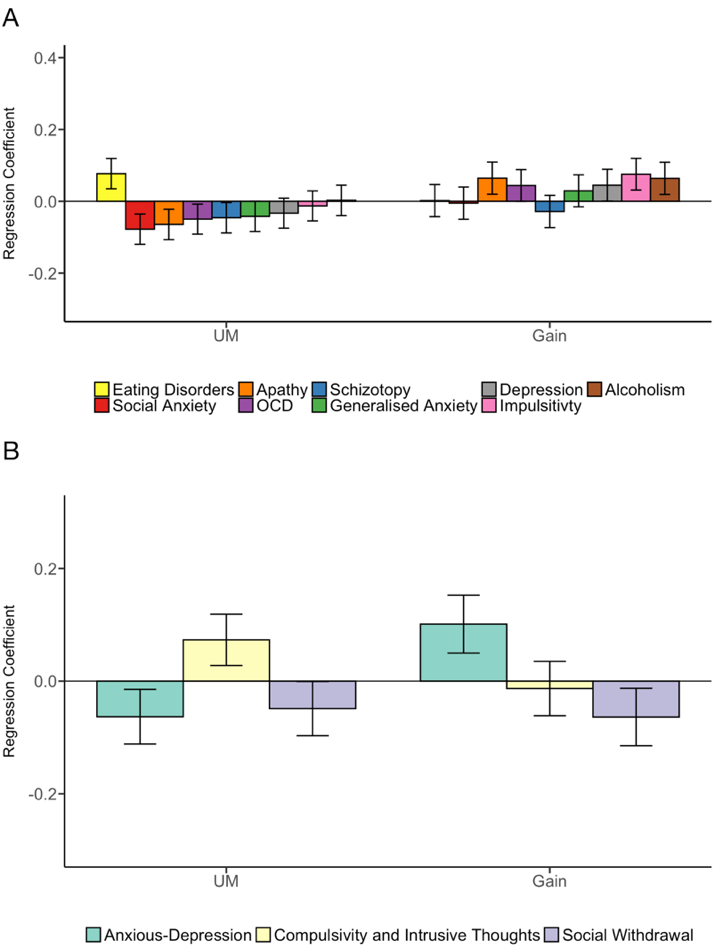


**Fig A. No significant relationships between psychiatric symptoms scores and model parameters.** **A**. Symptom scores from experiment 2 were entered into a multiple regression model predicting the model parameters. No significant association was obtained between individual symptom scores and model parameters, although qualitatively we again observed negative relationships between uncertainty modulation and several of the same symptom scores (e.g. depression and anxiety) that were significantly negative in experiment 1. **B**. Three latent factors based on symptoms scores from experiment 2 (see Methods) were entered into a multiple regression model predicting model parameters. These were Anxious-Depression (green), Compulsivity and Intrusive Thought (yellow), and Social Withdrawal (purple). No significant relationships between the factors and model parameters were observed. See Methods for details on the regression models. Error bars indicate s.e.m. All regressions results shown control for the influence of age, gender, and IQ.


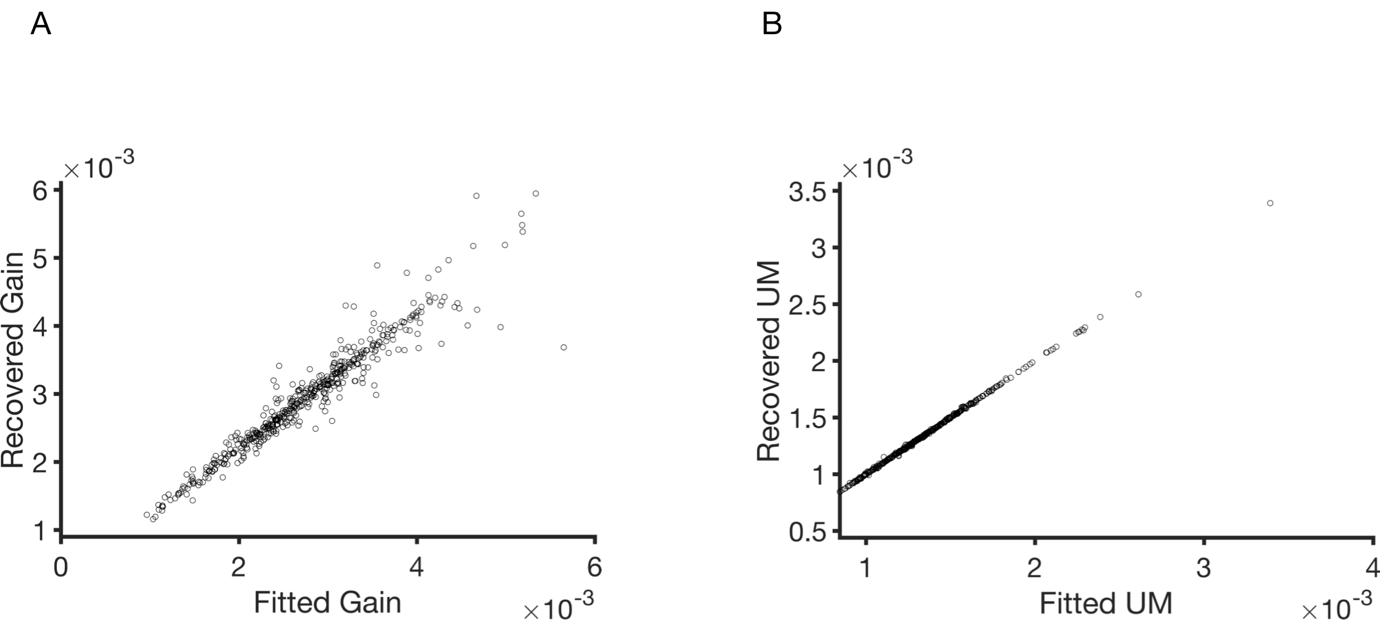


**Fig B.** **Results from parameter recovery simulations for experiment 1 for both (A) Gain and (B) UM**. Recovery simulations were done by simulating the fitted parameter for each participant, and then fitting to synthetically generated data (i.e. accuracy and mean response time). Both figures highlight a reasonably stable fit.


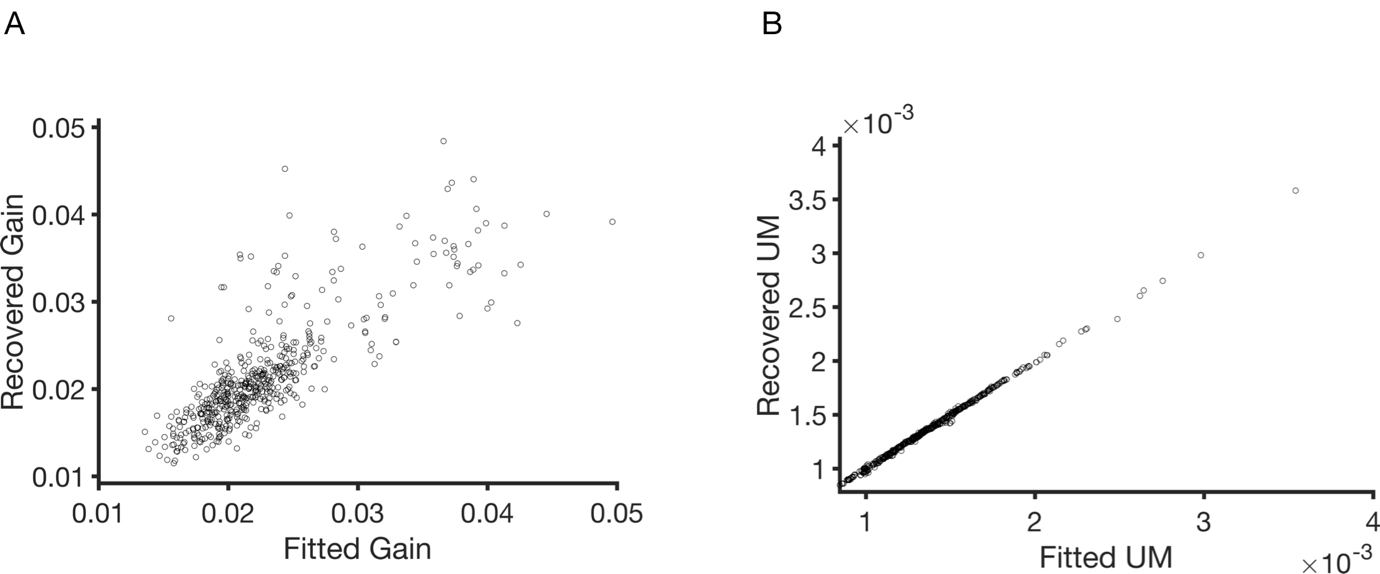


**Fig C.** **Results from parameter recovery simulations for experiment 2 for both (A) Gain and (B) UM**. Gain recovery for experiment 2 fits is more challenging, which could be partly due to the lower variance in difficulty experienced by each participant in experiment 2 (See Fig D).


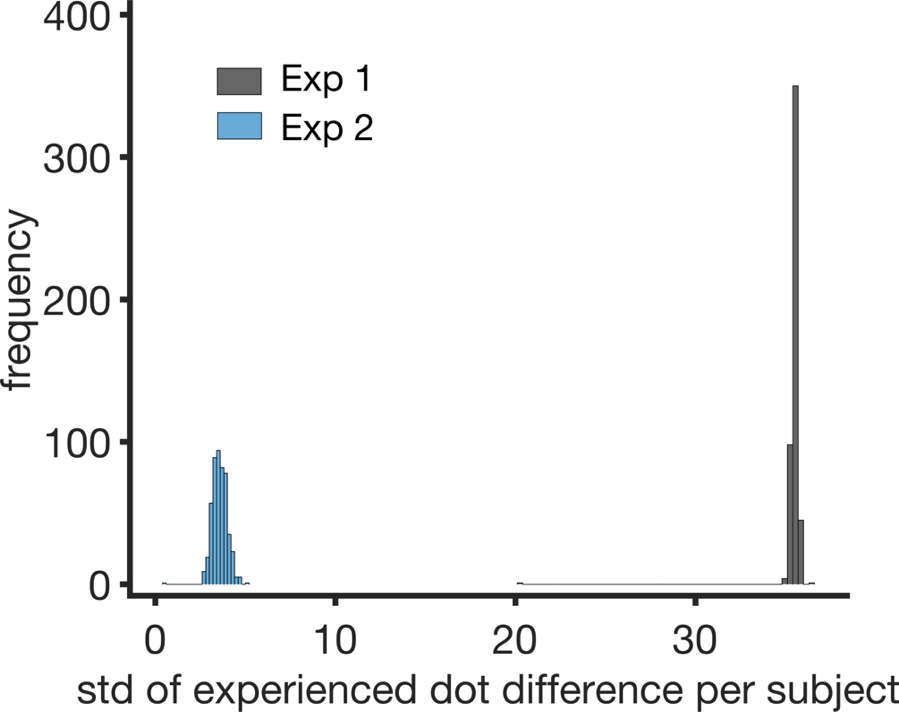


**Fig D. Variance in difficulty experienced by participants in Experiment 1 and 2.** Distribution of standard deviation of experienced dot difference per subject for experiment 1 (black) and experiment 2 (blue). Variance in difficulty between participants is much lower in experiment 2 compared to experiment 1 due to the use of a staircase procedure in the former.

1. Condon DM, Revelle W. The international cognitive ability resource: Development and initial validation of a public-domain measure. Intelligence. 2014;43:52-64. doi: 10.1016/j.intell.2014.01.004.
