# Supplementary material for: Explaining distortions in metacognition with an attractor network model of decision uncertainty
Source: PLoS Comput Biol. 2021 Jul 26;17(7):e1009201. doi: 10.1371/journal.pcbi.1009201 (PMC8341696; doi:10.1371/journal.pcbi.1009201)
Supplement: S3 Appendix — (DOCX) [file pcbi.1009201.s003.docx]

**S3 Appendix**

**Changes in metacognitive bias driven by UM**


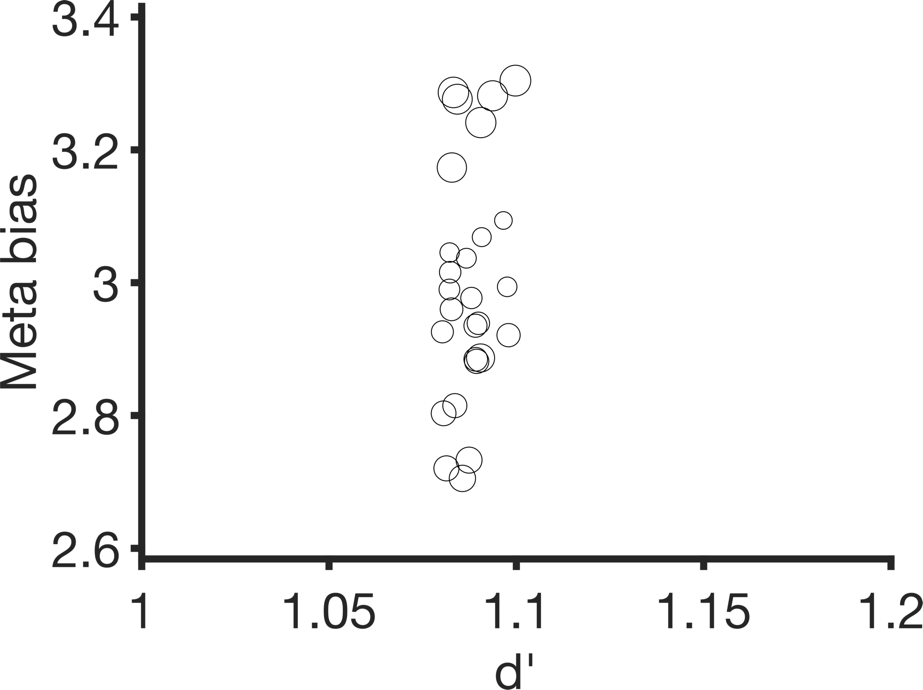


**Fig A.** **Changes in metacognitive bias are driven by changes in parameter governing higher-order nodes of the circuit (UM parameter) independently of changes in performance (**$\boldsymbol{d'}$**)**. Each circle is a mean of 50 simulations. For each circle, the same dot difference (2.8 in log space) and gain parameter (0.025) were used, but UM was varied between 0.002 and 0.006. Size of circle indicates UM value (larger circle = larger value).

More specifically, we simulated our model under a fixed difficulty level (dot difference set at 2.8 in log space), thereby maintaining a constant d’. We then examined the effect of 27 UM different parameter values spaced between 0.02 – 0.06, while fixing the gain parameter at 0.025 (as in our other analyses, e.g. Fig 2 in the main manuscript). Each simulation was repeated 50 times. Our results show that, in this particular subspace of the model parameters, UM has a strong effect on mean confidence but not d’.

In [1], the authors reproduce a similar result – matched d’ but introducing changes in confidence – by computing confidence primarily from the ‘less normalised units’ in their model. However, we would emphasise that the confidence bias we simulate here is likely distinct to that induced by positive evidence. More specifically, such changes in metacognitive bias here occur due to shifts in parameters governing higher-order nodes of the circuit (UM). We think that these two instances of confidence modulation in absence of d’ change may index different metacognitive “biases” in the system – at first-order and higher-order levels, respectively.

1. Maniscalco B, Odegaard B, Grimaldi P, Cho SH, Basso MA, Lau H, et al. Tuned inhibition in perceptual decision-making circuits can explain seemingly suboptimal confidence behavior. PLoS Comput Biol. 2021;17(3):e1008779-e. doi: 10.1371/journal.pcbi.1008779. PubMed PMID: 33780449.
