# Supplementary material for: Explaining distortions in metacognition with an attractor network model of decision uncertainty
Source: PLoS Comput Biol. 2021 Jul 26;17(7):e1009201. doi: 10.1371/journal.pcbi.1009201 (PMC8341696; doi:10.1371/journal.pcbi.1009201)
Supplement: S4 Appendix — (DOCX) [file pcbi.1009201.s004.docx]

**S4 Appendix**

**Experiment 1 fit with holdout**

To demonstrate that our model fits generalise to predict unseen test data, we re-ran our fitting algorithm on all Experiment 1 participants – holding out a 30% random non-stratified partition of each participant’s data for validation, and fitting on the remaining 70%.

Fig A below shows that the model generalises well in the case of choice accuracy (A) and response times (B).
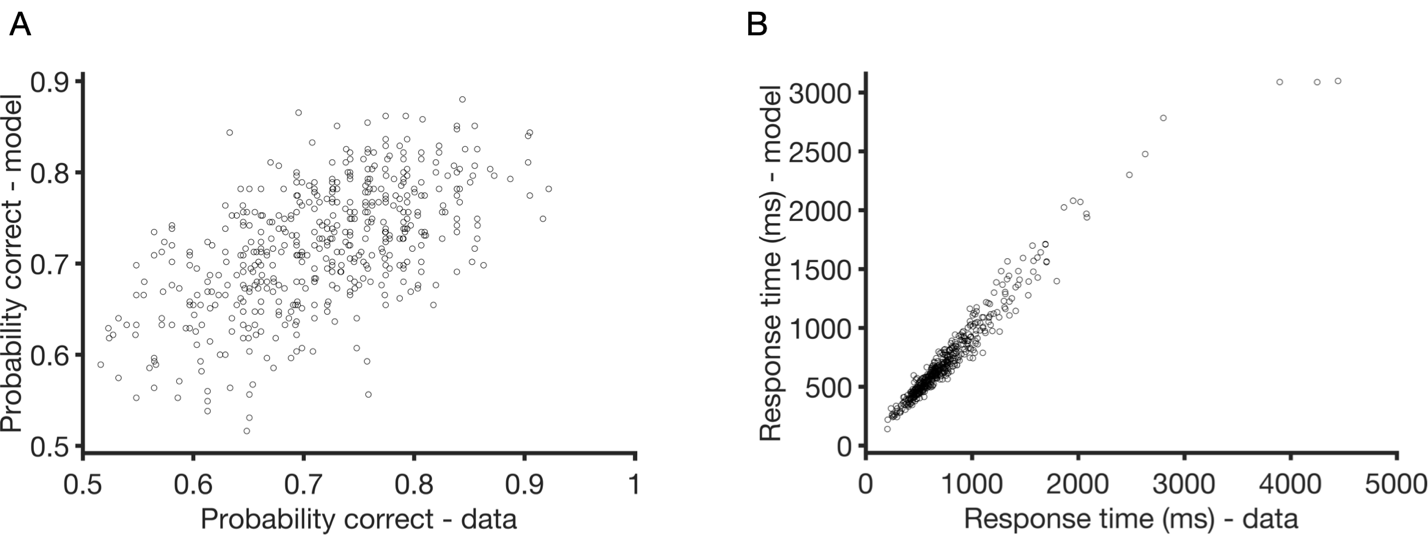


**Fig A.**  **Model parameters fitted to a subset (70%) of the data were then used to simulate data for each participant.** Empirical overall accuracy and mean response time were calculated from the holdout (30%) set unseen by the model during fitting**.** The model fits participants’ overall accuracy (A) and response time (B).
