# Supplementary material for: Explaining distortions in metacognition with an attractor network model of decision uncertainty
Source: PLoS Comput Biol. 2021 Jul 26;17(7):e1009201. doi: 10.1371/journal.pcbi.1009201 (PMC8341696; doi:10.1371/journal.pcbi.1009201)
Supplement: S5 Appendix — (DOCX) [file pcbi.1009201.s005.docx]

**S5 Appendix**

**Other supporting figures**


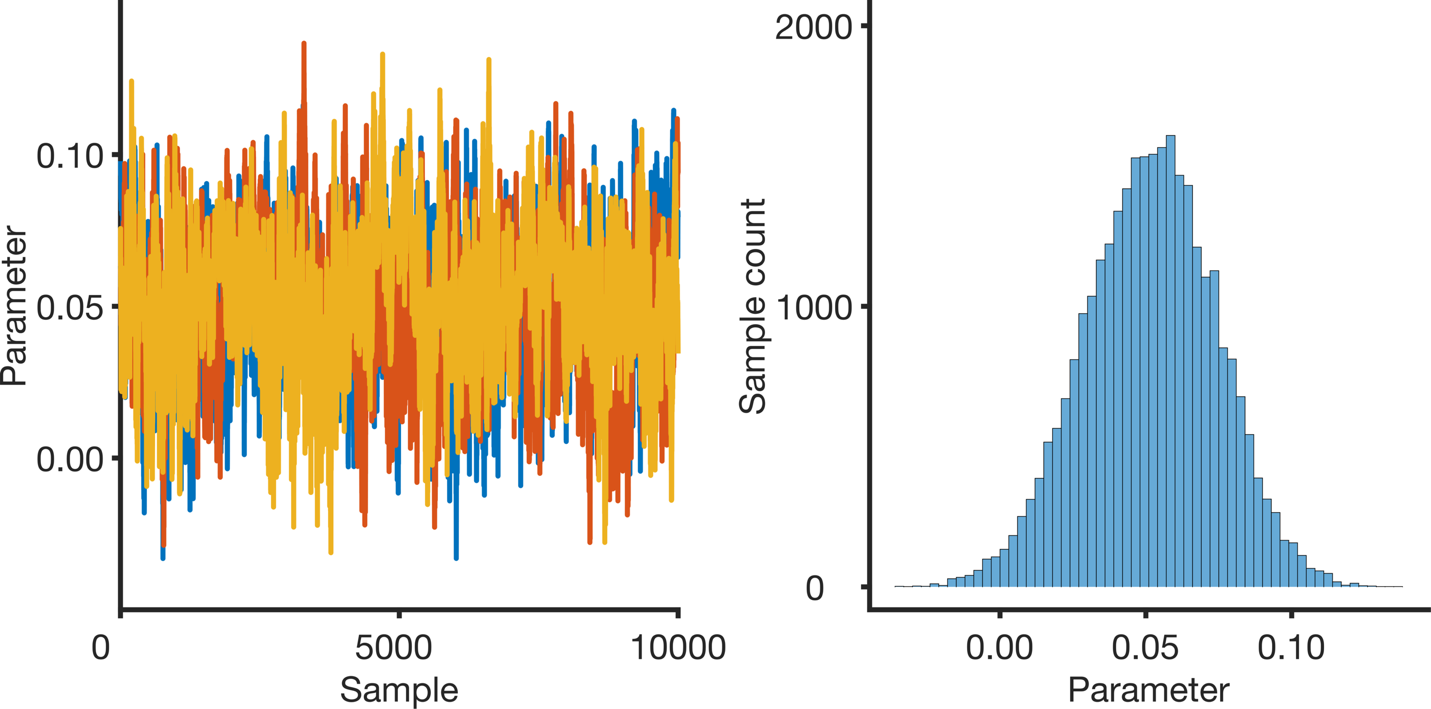


**Fig A. Hierarchical estimation of the impact of fitted UM on observed meta_d’/d’ ratio** **using a simultaneous regression approach with the UM parameter as a covariate (Harrison et al., 2020).** A positive association between the strength of uncertainty modulation and metacognitive efficiency (mean value of mu_beta = 0.0516, 95% highest density interval = (0.0813, 0.0016)).


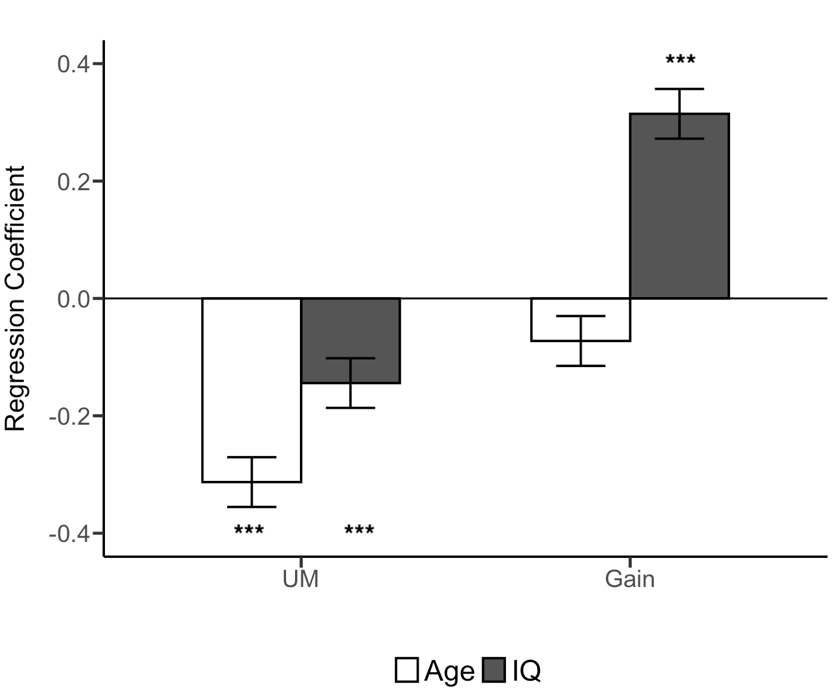


**Fig B.** **Relationship between Age/IQ and model parameters.** Age and IQ data from experiment 1 were entered into a multiple regression model (controlling for gender) predicting the strength of uncertainty modulation and gain parameters from the model fits to task performance (choices and response times). The results reveal that higher IQ is significantly associated with increased gain – suggesting a strong relationship between IQ and accuracy. The relationship is between IQ and Uncertainty Modulation (UM) is weaker, but still significant. Older age is associated with weaker UM.

**
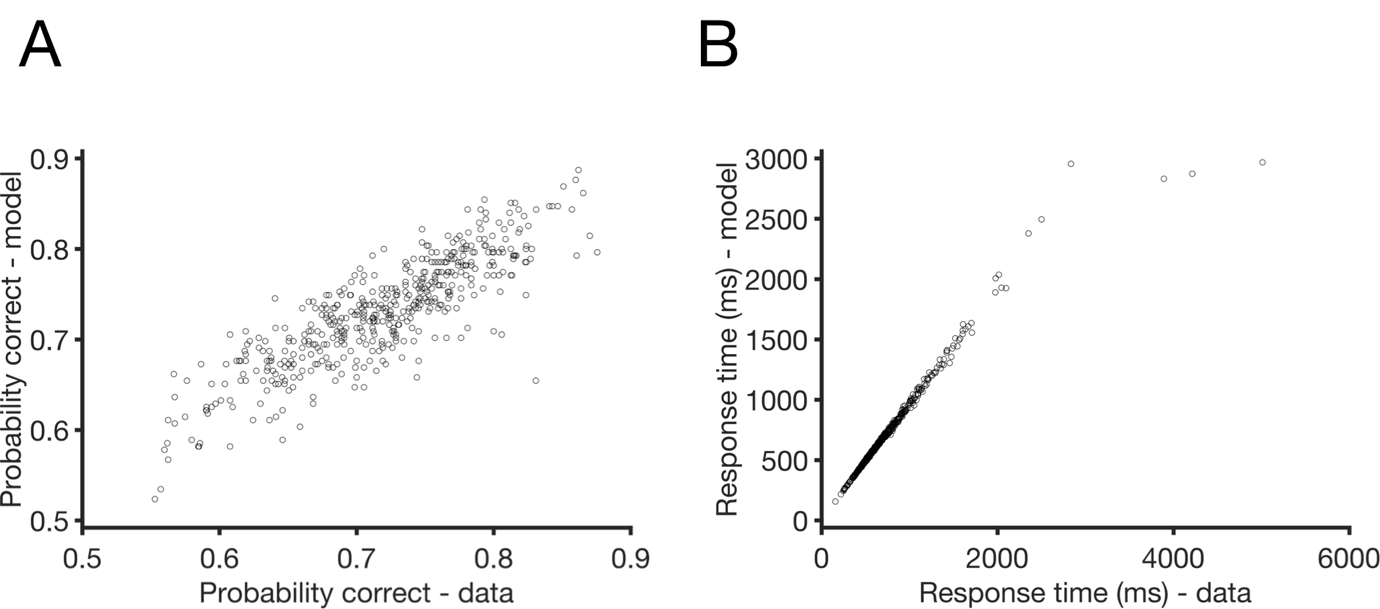
**

**Fig C. (A) Individual accuracy and (B) mean response time model fits for Experiment 1 without resetting the random number generator seed.**


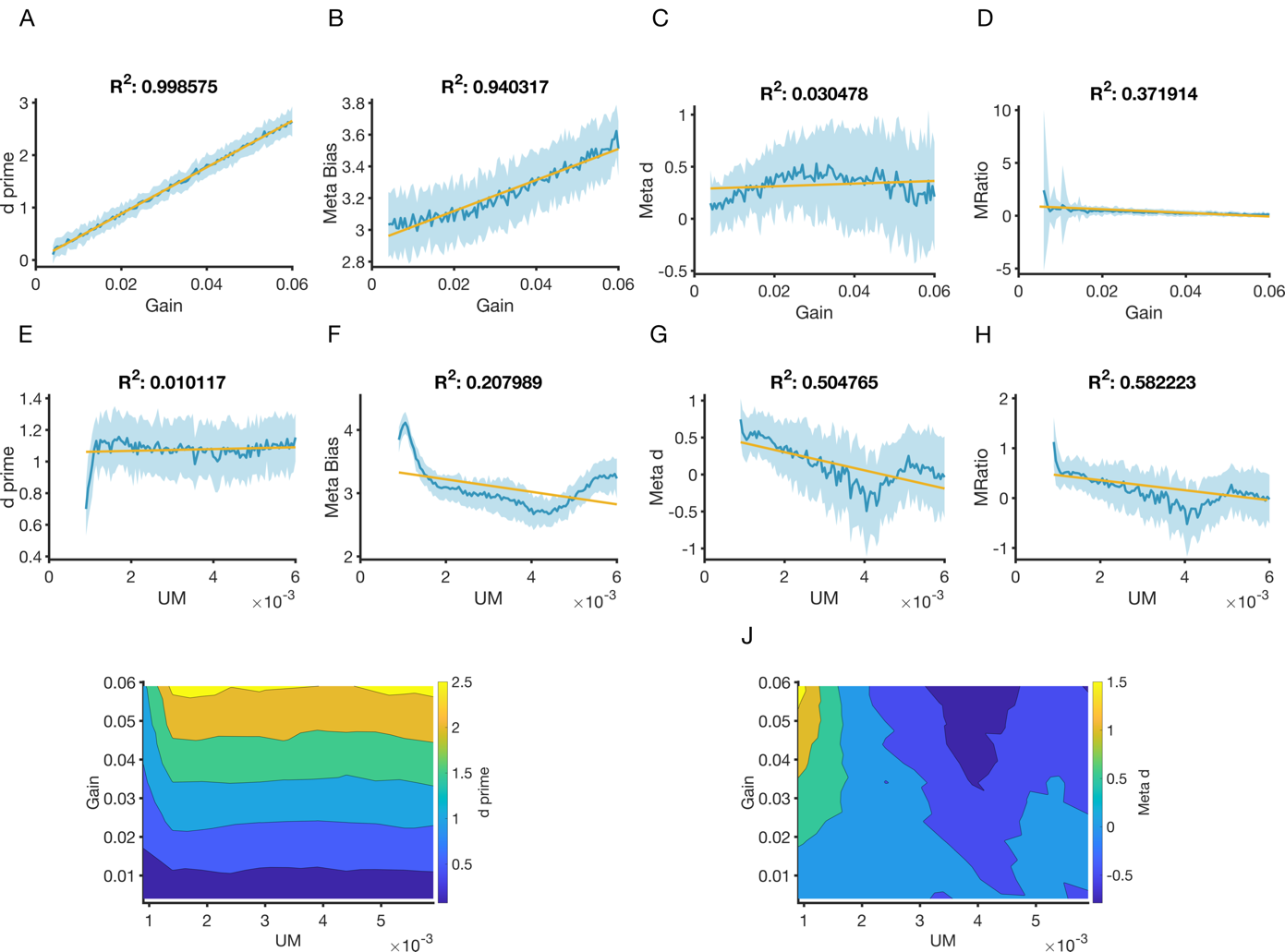


**Fig D.**  **Simulations of Figure 2 in the main manuscript with alternative parameter values.** In simulations (**A-H**), where the gain (UM) parameter is varied, UM (gain) was fixed at 0.0015 (0.0029). In this parameter subspace, meta_d’/d’ ratio is low – particularly in relation to d’.
