# Supplementary material for: Explaining distortions in metacognition with an attractor network model of decision uncertainty
Source: PLoS Comput Biol. 2021 Jul 26;17(7):e1009201. doi: 10.1371/journal.pcbi.1009201 (PMC8341696; doi:10.1371/journal.pcbi.1009201)
Supplement: S1 Text — (DOCX) [file pcbi.1009201.s006.docx]

**S1 Text**

**Exclusion Criteria**

Experiment 1

Participants were required to pass all of the following 6 tests to be included:

1. Performance of at least 55% correct
2. Prior to the task, a test to measure how well participants understood the confidence scale (what would you rate your confidence if you were sure your judgment was correct (incorrect)? Correct answer is 11 (1)
3. When rating pre- and post- global expected performance level: minimum expected performance level ≤ average performance level ≤ maximum expected performance level
4. A “catch” question: “If you are paying attention to these questions, please select ‘A little’ as your answer”
5. Not repeatedly selecting the same confidence rating across trials

Experiment 2 had the same exclusion criteria adopted – with the addition of prohibiting participants who took part in Experiment 1 from taking part in Experiment 2.
