# Supplementary material for: Explaining distortions in metacognition with an attractor network model of decision uncertainty
Source: PLoS Comput Biol. 2021 Jul 26;17(7):e1009201. doi: 10.1371/journal.pcbi.1009201 (PMC8341696; doi:10.1371/journal.pcbi.1009201)
Supplement: S2 Text — (DOCX) [file pcbi.1009201.s007.docx]

**S2 Text**

**Integration onset timing parameter**

In our model, we use an inhibitory mechanism to gate the integration of input in the uncertainty monitoring population. Such an inhibitory mechanism has been proposed to originate from a subcortical circuit. For example, the threshold crossing (response threshold in our model, which triggers top-down inhibition) could be detected by the superior colliculus via basal ganglia [1,2]. More complex gating pathways in the brain, including disinhibitory circuits, have been proposed to also involve subcortical structures, such as the basal ganglia and thalamus [3]. As a proxy for modelling such complex and extended neural networks, we instead modelled just the onset and offset of top-down inhibition. The onset of this top-down inhibition is assumed to have been learned e.g. through the basal ganglia (see [4]), via changes in the influence of neuromodulators [5]. In this sense, the exact onset time (mediated partially through gating and top-down inhibitory mechanisms) can be learned using models such as the one outlined by [6]. Providing an explicit account of such complex neural circuit dynamics is beyond the scope of this work, and we hope future work will address this in more detail.

The timing value (200ms) has been explored in detail in our previous work [7]. In this work, we decided to fix this value across all subjects and vary only the other two parameters.

1. Lo C-C, Wang X-J. Cortico–basal ganglia circuit mechanism for a decision threshold in reaction time tasks. Nat Neurosci. 2006;9(7):956-63.

2. Crapse TB, Sommer MA. Frontal eye field neurons with spatial representations predicted by their subcortical input. Journal of Neuroscience. 2009;29(16):5308-18.

3. Wang X-J, Yang GR. A disinhibitory circuit motif and flexible information routing in the brain. Current opinion in neurobiology. 2018;49:75-83.

4. Hazy TE, Frank MJ, O'reilly RC. Towards an executive without a homunculus: computational models of the prefrontal cortex/basal ganglia system. Philosophical Transactions of the Royal Society B: Biological Sciences. 2007;362(1485):1601-13.

5. Frank MJ. Dynamic dopamine modulation in the basal ganglia: a neurocomputational account of cognitive deficits in medicated and nonmedicated Parkinsonism. Journal of cognitive neuroscience. 2005;17(1):51-72.

6. Alexander WH, Brown JW. Medial prefrontal cortex as an action-outcome predictor. Nat Neurosci. 2011;14(10):1338-44.

7. Atiya NAA, Rañó I, Prasad G, Wong-Lin K. A neural circuit model of decision uncertainty and change-of-mind. Nat Commun. 2019;10(1):2287-. doi: 10.1038/s41467-019-10316-8. PubMed PMID: 31123260.
