# Supplementary material for: Explaining distortions in metacognition with an attractor network model of decision uncertainty
Source: PLoS Comput Biol. 2021 Jul 26;17(7):e1009201. doi: 10.1371/journal.pcbi.1009201 (PMC8341696; doi:10.1371/journal.pcbi.1009201)
Supplement: S3 Text — (DOCX) [file pcbi.1009201.s008.docx]

**S3 Text**

**UM as a low dimensional marker of metacognitive profile**

We have run a regression where mean empirical confidence for Experiments 1 and 2 was entered as a predictor of fitted UM, controlling for gender, IQ and age. For experiment 2, mean empirical confidence was indeed positively correlated with UM, despite the distribution matching approach (Beta = 0.12, SEM = 0.04, p<0.005). A similar positive correlation between UM and mean empirical confidence was observed in Experiment 1 (Beta = 0.0017, SEM = 0.04), but this did not reach significance (p>0.5). Overall, these findings suggest that such fitted parameters may provide insight into a subjects’ metacognitive profile even in the absence of confidence rating data.
